# Toll-like receptor 4 signaling in trigeminal ganglion neurons contributes tongue-referred pain associated with tooth pulp inflammation

**DOI:** 10.1186/1742-2094-10-139

**Published:** 2013-11-23

**Authors:** Kinuyo Ohara, Kohei Shimizu, Shingo Matsuura, Bunnai Ogiso, Daisuke Omagari, Masatake Asano, Yoshiyuki Tsuboi, Masamichi Shinoda, Koichi Iwata

**Affiliations:** 1 Department of Endodontics, Nihon University School of Dentistry, 1-8-13 Kandasurugadai, Chiyoda-ku, Tokyo 101-8310, Japan; 2 Divisions of Advanced Dental Treatment, Dental Research Center, Nihon University School of Dentistry, Tokyo 101-8310, Japan; 3 Department of Pathology, Nihon University School of Dentistry, 1-8-13 Kandasurugadai, Chiyoda-ku, Tokyo 101-8310, Japan; 4 Division of Immunology and Pathobiology, Dental Research Center, Nihon University School of Dentistry, Tokyo 101-8310, Japan; 5 Department of Physiology, Nihon University School of Dentistry, 1-8-13 Kandasurugadai, Chiyoda-ku, Tokyo 101-8310, Japan; 6 Division of Functional Morphology, Dental Research Center, Nihon University School of Dentistry, Tokyo 101-8310, Japan; 7 Division of Applied System Neuroscience Advanced Medical Research Center, Nihon University Graduate School of Medical Science, 30-1 Ohyaguchi-Kamimachi Itabashi, Tokyo 173-8610, Japan

**Keywords:** Tooth-pulp inflammation, Tongue-referred pain, Trigeminal ganglion, Heat shock protein

## Abstract

**Background:**

The purpose of the present study is to evaluate the mechanisms underlying tongue-referred pain associated with tooth pulp inflammation.

**Method:**

Using mechanical and temperature stimulation following dental surgery, we have demonstrated that dental inflammation and hyperalgesia correlates with increased immunohistochemical staining of neurons for TLR4 and HSP70.

**Results:**

Mechanical or heat hyperalgesia significantly enhanced in the ipsilateral tongue at 1 to 9 days after complete Freund’s adjuvant (CFA) application to the left lower molar tooth pulp compared with that of sham-treated or vehicle-applied rats. The number of fluorogold (FG)-labeled TLR4-immunoreactive (IR) cells was significantly larger in CFA-applied rats compared with sham-treated or vehicle-applied rats to the molar tooth. The number of heat shock protein (Hsp) 70-IR neurons in trigeminal ganglion (TG) was significantly increased on day 3 after CFA application compared with sham-treated or vehicle-applied rats to the molar tooth. About 9.2% of TG neurons were labeled with DiI applied to the molar tooth and FG injected into the tongue, and 15.4% of TG neurons were labeled with FG injected into the tongue and Alexa-labeled Hsp70-IR applied to the tooth. Three days after Hsp70 or lipopolysaccharide (LPS) application to the tooth in naive rats, mechanical or heat hyperalgesia was significantly enhanced compared with that of saline-applied rats. Following successive LPS-RS, an antagonist of TLR4, administration to the TG for 3 days, the enhanced mechanical or heat hyperalgesia was significantly reversed compared with that of saline-injected rats. Noxious mechanical responses of TG neurons innervating the tongue were significantly higher in CFA-applied rats compare with sham rats to the tooth. Hsp70 mRNA levels of the tooth pulp and TG were not different between CFA-applied rats and sham rats.

**Conclusions:**

The present findings indicate that Hsp70 transported from the tooth pulp to TG neurons or expressed in TG neurons is released from TG neurons innervating inflamed tooth pulp, and is taken by TG neurons innervating the tongue, suggesting that the Hsp70-TLR4 signaling in TG plays a pivotal role in tongue-referred pain associated with tooth pulp inflammation.

## Background

It is well known that orofacial dysesthesia or referred pain sometimes occurs as a secondary hyperalgesia associated with tooth pulpal inflammation [1]. A number of previous studies have reported that the orofacial persistent pain following trigeminal nerve injury or orofacial inflammation is known to cause various motor as well as sensory disorders in the orofacial regions such as masticatory dysfunction and/or swallowing disorder [2]. It has also been reported that persistent pain is sometimes developed in oral structures following tooth pulp (TP) inflammation in human subjects [3]. Although sensitization of the peripheral nervous systems (PNS) following pulpal inflammation is thought to be involved in pathogenesis of the referred pain in oral structures, the peripheral mechanisms underlying orofacial-referred pain associated with TP inflammation remain unclear. Since the presence of orofacial dysesthesia and referred pain often lead to serious problems such as misdiagnosis and inappropriate treatment [4], it is necessary to clarify the mechanisms underlying orofacial dysesthesia and referred pain associated with TP inflammation.

Bacterial byproducts and various chemical mediators induced by peripheral infection or inflammation activate nociceptors in primary afferent neurons, and a barrage of action potentials are generated in primary afferent neurons which are sent to the central nervous system (CNS), resulting in the sensitization of nociceptive neurons in the PNS [5]. Substance P or calcitonin gene-related peptide is known to be released from trigeminal ganglion (TG) neurons following temporomandibular joint inflammation and these neuropeptides affect the excitability of adjacent TG neurons innervating the non-inflamed facial skin [6]. These findings strongly suggest that neuron-neuron interaction in the TG is involved in modulation of TG neurons innervating non-inflamed oral structures following TP inflammation.

Toll-like receptors (TLRs) are known as transmembrane pattern-recognition receptors that initiate signals in response to diverse pathogen-associated molecular patterns (PAMPs). After tissue injury or cellular stress, TLRs detect endogenous ligands known as danger-associated molecular patterns (DAMPs) [7]. It is well known that TLRs in primary sensory neurons, such as dorsal root ganglion (DRG) and trigeminal ganglion (TG) neurons, are involved in the modulation of neuronal excitation, in particular, primary sensory neurons expressing TLR4 and TLR7 to sense exogenous PAMPs and endogenous DAMPs released after tissue injury or cellular stress [8]. These neuronal TLRs are thought to be involved in the development of pathological pain following peripheral inflammation.

Heat shock protein 70 (Hsp70) is well known as a specific ligand for TLRs and has been reported to be expressed in the brain [9] and heart [10], and is involved in pathological pain associated with tissue injury or inflammation [11]. Previous studies have also reported that Hsp70 is expressed in the dental pulp following pulpal trauma or inflammation [12], therefore it is highly possible that Hsp70 is involved in the development of pathological pulpal pain and orofacial referred pain.

Together, we hypothesized that Hsp70 was expressed in the pulpal tissue or TG neurons after the TP inflammation and affected the excitability of TG neurons through TLR4 signaling, and played a crucial role in the development of tongue-referred pain. To test this hypothesis, we analyzed mechanical- and heat-evoked nocifensive reflex, TLR4 expression in TG after pulpal inflammation, primary afferent tracing using fluorogold (FG) and DiI applied into the tongue and lower first molar tooth (M1) to study if the single TG neuron innervates both pulp and tongue, Hsp70 expression in the TG after pulpal inflammation, fluorescent labeled-Hsp70 expression in TG neurons via axonal flow following labeled Hsp70 injection into the left M1TP, TG neuronal activity, RT-PCR analysis of Hsp70 in the inflamed tooth pulp and TG, and the effect of TLR4 agonist and antagonist on the nocifensive reflex.

## Methods

### Animals

This study was approved by the Animal Experimentation Committee at Nihon University. All surgery and animal care were conducted in accordance with the National Institutes of Health Guide for the Care and Use of Laboratory Animals and the guidelines for Institutional Animal Care, and the guidelines of the International Association for the Study of Pain [13]. Male Sprague–Dawley rats (*n* = 177, Japan SLC, Shizuoka, Japan) weighing 250 to 350 g were used in this study. The animals were maintained in a temperature-controlled room (23°C) with a 12/12-h light/dark cycle. Food and water were freely available.

### CFA application to the TP

Rats were lightly anesthetized with 2% isoflurane (Mylan, Canonsburg, PA, USA) and then deeply anesthetized with an intraperitoneal (i.p.) application of sodium pentobarbital (50 mg/kg; Schering Plough, Whitehouse Station, NJ, USA). Then the rats were placed on a warm mat (37°C) in the supine position to allow for the application of CFA (Sigma-Aldrich, St. Louis, MI, USA). A total of 50% CFA (diluted in saline) or vehicle (isotonic saline) was applied to the M1 unilaterally. The rat’s mouth was gently opened and the left M1TP was exposed by means of a low-speed dental drill with a round tungsten carbide bur under water cooling. A small piece of dental paper point (diameter, 0.15 mm; length, 1.5 mm) soaked with CFA or vehicle was applied to the exposed M1TP. Then the exposed pulp cavity was sealed with dental cement.

### Head-withdrawal reflex threshold measurement

The head-withdrawal reflex threshold (HWT) to mechanical and heat stimulation of the lateral edge of tongue (3 mm posterior from tip of tongue) was measured on day 3 after saline or CFA application to the tooth pulp, under light anesthesia with 2% isoflurane (Mylan, Canonsburg, PA, USA) in oxygen. Bipolar enamel-coated stainless steel wire electrodes (Narishige, Tokyo, Japan) were placed in the splenius capitis muscle for electromyogram (EMG) recording of the reflex response (inter-electrode distance, 5 to 6 mm).

Lower jaw was gently pulled with plastic strings and rat’s mouth was kept open, and then mechanical stimulation (0 to 130 g; 10 g/s; cutoff, 130 g) was applied to the lateral edge of the tongue ipsilateral to the CFA or vehicle application by using forceps with flat tips (4 mm^2^; Panlab s.l., Barcelona, Spain) in lightly anesthetized rats (*n* = 7 in each group). The stimulus velocity was manually controlled consecutively from 0 g to threshold values at a speed of approximately 10 g/s. The threshold intensity for evoking EMG activity by mechanical stimulation of the tongue was defined as the mechanical HWT.

Heat stimulation (35-60°C; 1°C/s; cutoff, 60°C) was also applied to the lateral edge of the tongue ipsilateral to the CFA or vehicle application by using a contact heat probe (9 mm^2^; Intercross, Tokyo, Japan) in lightly anesthetized rats (*n* = 7 in each group). The threshold temperature for evoking EMG activity by heat stimulation to the tongue was defined as the heat HWT. The mechanical or heat stimulation was applied three times with 5-min intervals, and the mean value of the HWTs was calculated. The splenius capitis EMG was recorded following mechanical and heat stimulation of the tongue on days 1, 3, 5, 7, 9, 11, 14, 21, 28 (days 21, 28 data not shown.). Time-course change in mean HWT value to mechanical or heat stimulation of the ipsilateral tongue was measured in CFA, vehicle, or sham rats. The baseline HWT value to mechanical or heat stimulation was measured before CFA or vehicle application, or sham treatment of the tooth.

### TLR4 immunohistochemistry in combination with FG tracer

For TLR4 immunohistochemistry in combination with FG tracer into tongue, 5.0 μL of 10% FG (Wako) dissolved in saline was applied into the lateral edge of the tongue of the rats anesthetized with 2% isoflurane. On day 3 after FG application, rats were lightly anesthetized with 2% isoflurane and then deeply anesthetized with sodium pentobarbital (50 mg/kg, i.p.) for the application of CFA or vehicle into the M1TP. On day 3 after CFA or vehicle application, rats were transcardially perfused with saline, followed by a fixative containing 4% paraformaldehyde in 0.1 M phosphate buffer (pH 7.4) under same anesthesia used for CFA or vehicle application (*n* = 5 in each group). TGs in the ipsilateral side to CFA, vehicle, or sham operation were dissected out after perfusion and post-fixed in 4% PFA for 1 day at 4°C. The specimens were then transferred to 20% sucrose (w/v) in distilled water for several days for cryopreservation, were then embedded in Tissue Tek (Sakura Finetek, Torrance, CA, USA), and stored until cryosectioning at −20°C. TG sections of 10 μm were cut in the horizontal plane along the longitudinal axis. Every eighth section was thaw-mounted on MAS-GP microslide glass (Matunami, Osaka, Japan) and dried overnight at room temperature. Four sections were chosen from each TG in each rat and these were processed for TLR4 immunohistochemistry. Sections were incubated with rabbit anti-TLR4 polyclonal antibody (1:200; abcam) after dilution at a concentration of 1:800 in 0.01 M PBS containing 4% normal goat serum (NGS) and 0.3% Triton X-100 (Sigma-Aldrich) on day 3 at 4°C. After rinsing with 0.01 M PBS, sections were incubated in Alexa Fluor 488 goat anti-rabbit IgG (1:200 in 0.01 M PBS; Invitrogen, Paisley, UK) for 2 h at room temperature. After rinsing with 0.01 M PBS, sections were cover-slipped in mounting medium (Thermo Fisher Scientific, Fremont, CA, USA) and examined under a fluorescence microscope and analyzed using a BZ-9000 system (Keyence, Osaka, Japan). No specific labeling was observed in the absence of primary antibody. The number of TLR4-IR cells in TG in the V3 branch region were analyzed and counted in each rat (SensiveMeasure; Mitani, Fukui, Japan; *n* = 5 in each group). In addition, the relative numbers of them were calculated by the following formula: 100 × number of neurons for TLR4- or FG-IR cells/FG-IR cells.

### Hsp70 immunohistochemistry

For Hsp70 immunohistochemistry, sections were incubated with rabbit anti-Hsp70 polyclonal antibody (1:200; abcam) after dilution at a concentration of 1:800 in 0.01 M PBS containing 4% NGS and 0.3% Triton X-100 (Sigma-Aldrich) on day 3 at 4°C. After rinsing with 0.01 M PBS, sections were incubated in Alexa Fluor 488 goat anti-rabbit IgG (1:200 in 0.01 M PBS; Invitrogen, Paisley, UK) for 2 h at room temperature. After rinsing with 0.01 M PBS, sections were cover-slipped in mounting medium (Thermo Fisher Scientific, Fremont, CA, USA) and examined under a fluorescence microscope and analyzed using a BZ-9000 system (Keyence, Osaka, Japan). No specific labeling was observed in the absence of primary antibody. The number of Hsp70-IR cells in TG in the V3 branch region were analyzed and counted in each rat (SensiveMeasure; Mitani, Fukui, Japan; *n* = 5 in each group). In addition, the relative numbers of them were calculated by the following formula: 100 × number of Hsp70-IR cells/all TG neurons.

### Labeling of recombinant Hsp70

The Alexa Fluor 594 labeling of Hsp70 (R&D Systems) was performed with Alexa Fluor 594 microscale protein labeling kit (Molecular Probes). Briefly, 50 μg of Hsp70 was mixed with 5.0 μL of 1 M sodium bicarbonate and pipetted thoroughly. The sample was further mixed with 2.6 μL of Alexa Fluor 594 solution and incubated for 15 min at room temperature. The reaction mixture was applied to the spin column and unreacted dye was eliminated. The flowthrough was collected and the protein concentration was measured. The labeled protein was aliquoted and stored at −80°C until use.

### FG, DiI, and recombinant Hsp70 application to the pulp

For FG, DiI, and labeled recombinant Hsp70 application to the pulp, rats were deeply anesthetized with sodium pentobarbital (50 mg/kg, i.p.) and 5.0 μL of 10% FG dissolved in saline was applied into the lateral edge of the tongue. On day 4 after FG application, DiI (Invitrogen) saturated in 100% ethanol or labeled recombinant Hsp70 was also applied into M1 with paper point. Subsequently on day 3, rats were deeply anesthetized and perfused, and then TG was removed and sectioned. FG- and/or recombinant Hsp70-labeled cells, or FG- and/or DiI-labeled cells were studied in TG under fluorescent microscopy, respectively.

### Hsp70 or LPS application to the M1TP

Rats were anesthetized with sodium pentobarbital (50 mg/kg, i.p.), the recombinant Hsp70 or LPS was applied to the left M1TP with the same procedures as CFA application to the M1TP. On day 3 after drug application HWT to mechanical and heat stimulation of the tongue was measured. Saline was also administered as vehicle control.

### LPS-RS administration into TG

Rats were anesthetized, and a small hole (diameter, 1 mm) was drilled in the skull above V1/V2 and V3 branch of TG. The guide cannula was extended into the hole 9 mm below the skull surface into TG and was fixed to the skull with three stainless-steel screws and dental resin, according to the method by Katagiri et al. [14]. CFA- rats were administered saline (0.5 μL) or TLR4 antagonist LPS-RS (0.1 mM 0.5 μL/day; Invivo Gen) dissolved in saline (*n* = 5 in each group), and naive rats were administrated saline (0.5 μL) or LPS-RS (0.5 μL/day; Invivo Gen) dissolved in saline (*n* = 5 in each group) once a day into TG for 3 successive days (day 0 through day 2) (n = 5 in each group). HWTs were then measured on day 3 day under light anesthesia with 2% isoflurane in oxygen.

### Single neuron recording from TG neurons

Three days after CFA application to the M1TP or sham treatment of the M1 teeth, rats were anesthetized with pentobarbital-Na (50 mg/kg, i.p.). Trachea and femoral vein were cannulated for artificial respiration and intravenous administration of drugs, and then rats were fixed in the stereotaxic flame. Brain tissue over the TG was removed and TG surface was exposed, and the enamel-coated tungsten electrodes were inserted into the TG and single neuronal activities were recorded. During the recording session, rats were immobilized with pancuronium bromide (0.6 mg/kg/h, i.v. Schering-Plough, Darmstadt, Germany) and artificially ventilated, and end-tidal CO_2_ concentration and body temperature were maintained at 3.5 to 4.5% and at 37°C by a feedback-controlled heating blanket (Nihon koden, Tokyo, Japan). We used gentle brush and pressure stimuli as the search stimuli. When neuronal activity was obtained, brush, pressure, or noxious pinch (50 g) was applied to the receptive fields for 5 s with camel brush, brunt forceps, or small arterial clip (50 g), respectively. Spontaneous activity was recorded for 1 min before application of brush or pressure stimulus.

### Real-time PCR

Total RNA was purified using an RNeasy mini kit (QIAGEN, Tokyo, Japan). One μg of total RNA was subjected to first-strand cDNA synthesis with Superscript III reverse transcriptase (Life Technologies, Carlsbad, CA, USA), as previously described [15]. Real-time PCR was performed using LightCycler ® Nano (Roche, Tokyo, Japan) with SYBR green (TaKaRa, Tokyo, Japan). The Hsp70 and GAPDH primers were purchased from TaKaRa.

### Statistical analysis

Data were expressed as means ± SEM. Statistical analyses were performed by student’s *t*-test, or one-way ANOVA or two-way repeated-measures ANOVA followed by Bonferroni’s multiple comparison tests where appropriate. A value of *P* <0.05 was considered as significant.

## Results

### Nocifensive reflex to mechanical or heat stimulation of the tongue

**Figure 1:**
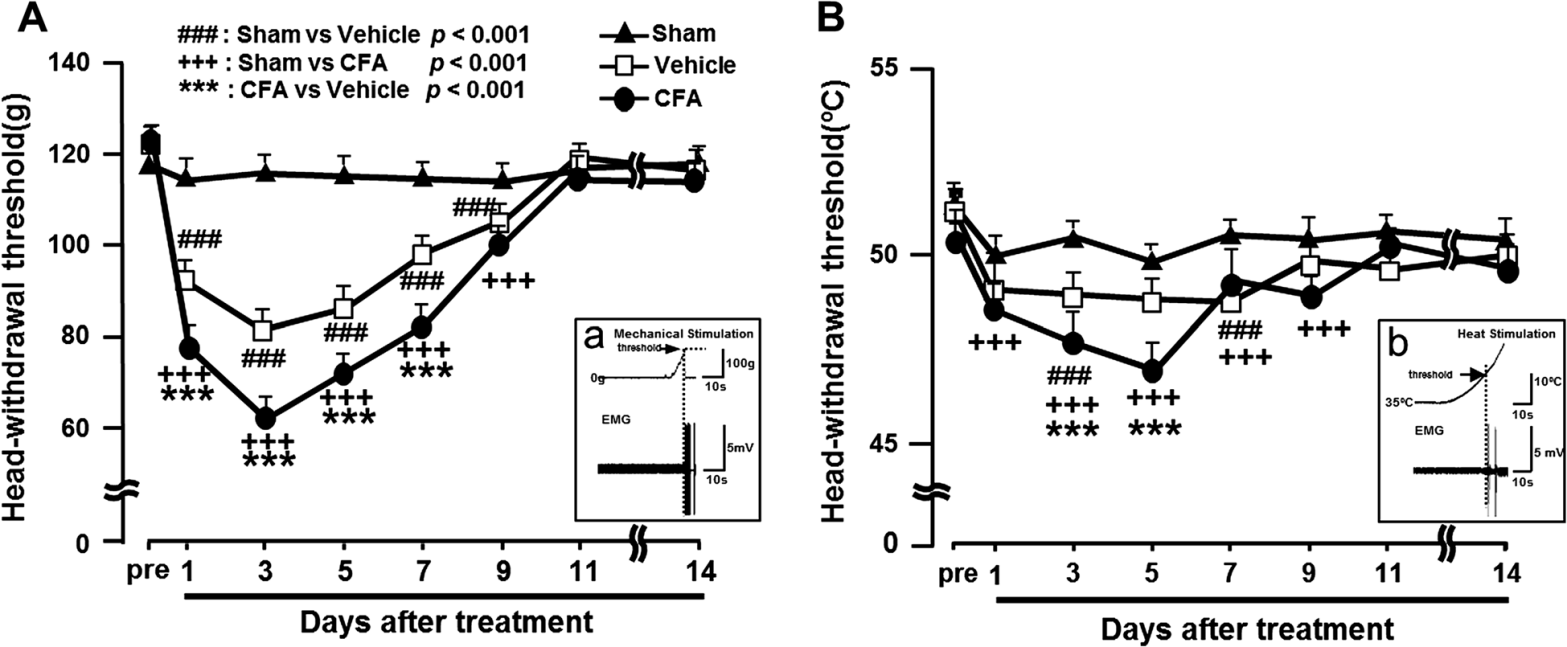
**Tongue mechanical/thermal hypersensitivity following CFA application to the tooth pulp in lightly anesthetized rats. (A)** Head-withdrawal threshold (HWT) to mechanical stimulation of the tongue. **(B)** HWT to heat stimulation of the tongue. Aa and Bb: EMG recordings from trapezius muscles during mechanical or heat stimulation of the tongue. ###: *P* < 0.001 (sham *vs*. vehicle), +++: *P* < 0.001 (sham *vs*. CFA), ***: *P* < 0.001 (CFA *vs*. vehicle).

The HWT to mechanical or heat stimulation of the ipsilateral tongue significantly decreased at 1 to 9 days after CFA application to the M1TP compared with that of sham-treated or vehicle-applied rats (mechanical, *P* < 0.001; heat, *P* < 0.001; *n* = 7 in each group) (Figure 1A: mechanical, B: heat). We also observed significant decrease in the head-withdrawal threshold to mechanical stimulation of the tongue in vehicle-applied rats at 1 to 9 days after vehicle application compared with sham rats (*P* < 0.001), and on days 3 and 7 heat head-withdrawal threshold was significantly lower in vehicle-applied rats compared to sham rats (*P* < 0.001). Sham-treated rats did not show any changes in HWT to mechanical or heat stimulation of the tongue.

### TLR4 expression in TG neurons

**Figure 2:**
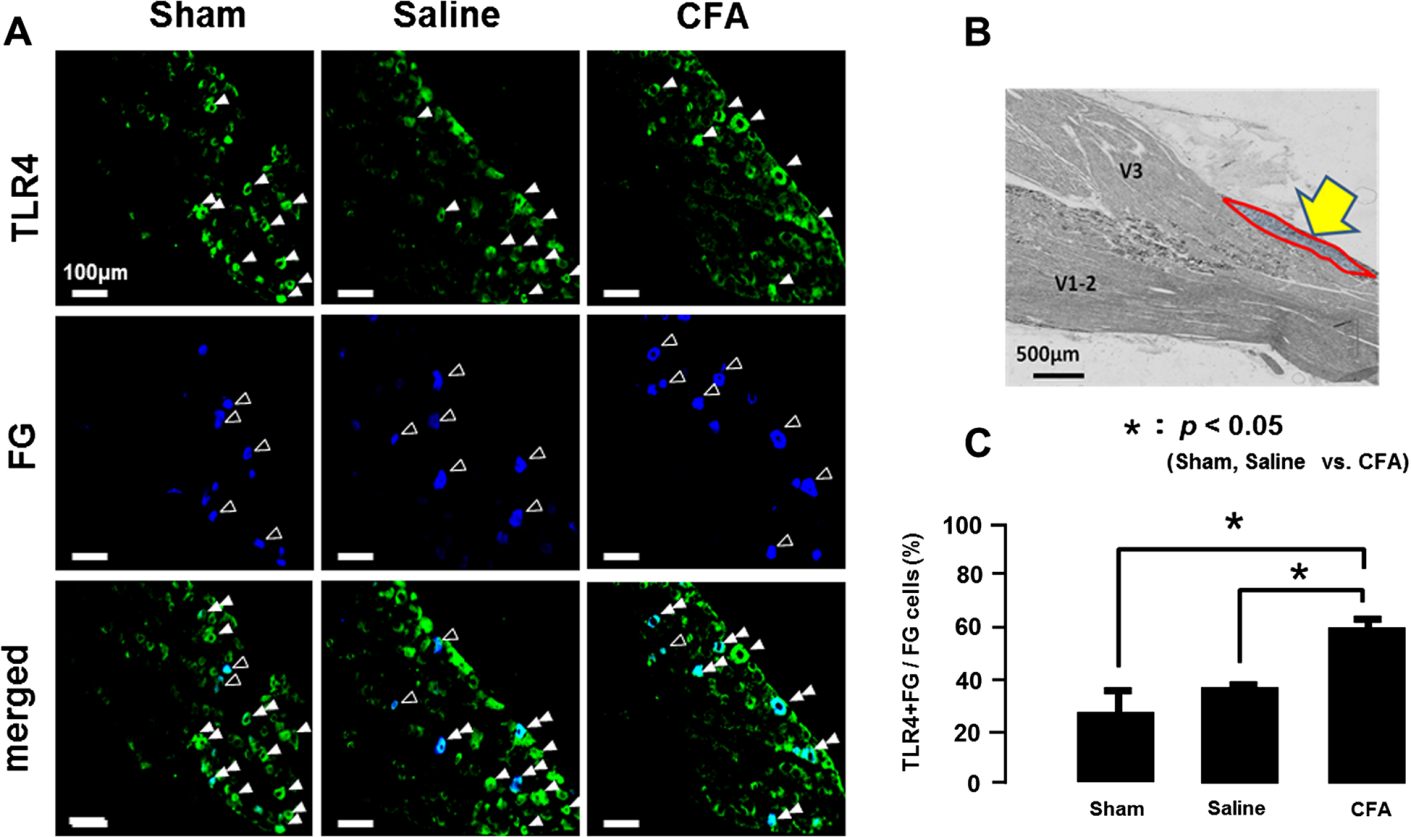
**TLR4 expression in TG neurons innervating tongue on day 3 after CFA injection into tooth pulp. (A)** TLR4-IR cells and FG-labeled cells in the TG in sham, saline-applied, or CFA-applied rats, respectively, and merged indicates TLR4-IR cells were merged with FG-labeled cells. FG was injected into the tongue. **(B)** Low magnification photomicrographs of the TG. **(C)** The mean number of TLR4-IR cells labeled with FG in sham, saline-applied, or CFA-applied rats. *: *P* <0.05.

To study if TG cells innervating the tongue show TLR4-IR following CFA application to M1TP, FG was injected into the tongue and then CFA was applied to the M1TP. Following CFA application to the M1TP, many TG neurons showed TLR4-IR, and some of them were retrogradely labeled with FG injected into the tongue (Figure 2A). The number of FG-positive TLR4-IR TG neurons was counted in the V3 branch region (the area indicated by the arrow in Figure 2B). The mean relative number of FG-labeled TLR4-IR cells was significantly increased in M1TP CFA-applied rats compared with M1TP sham and vehicle-applied rats (*n* = 5 in each group) (Figure 2C).

### Hsp70 expression following M1TP inflammation

**Figure 3:**
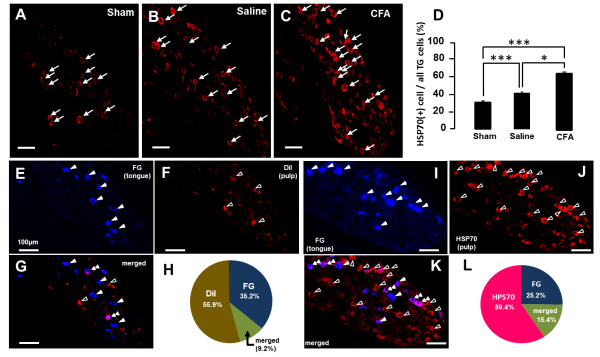
**Hsp70 expression in the TG neurons in sham, saline-applied, or CFA-applied rats, and FG-, DiI-, or Hsp70-labeled cells in the TG. A**, **B**, **C**: Hsp70-IR cells in the TG in sham **(A)**, saline-applied **(B)**, or CFA-applied rats **(C)**. **(D)** The mean number of Hsp70-IR cells in the TG in sham, saline-applied or CFA-applied rats. **E**, **F**, **G**: FG- **(E)** and DiI-labeled cells **(F)** or Alexa-labeled FG-labeled cells merged with DiI-labeled cells **(G)** in the TG. **(H)**: The pie graph indicates the percentage of DiI, FG, or merged cells in the TG. **I**, **J**, **K**: FG- **(I)** and Alexa-labeled Hsp70-labeled cells **(J)** or FG-labeled cells merged with HSP70-labeled cells **(K)** in the TG. **(L)** The pie graph indicates the percentage of FG-, Alexa-labeled Hsp70, or merged cells in the TG. Note that intra-pulpal Hsp70 was axonally transported to TG on day 3 after Alexa-labeled Hsp70 injection into the tooth pulp. *: *P* < 0.05, ***: *P* < 0.001.

A small number of Hsp70-IR cells were observed in TG in sham- and vehicle- applied rats, whereas a larger number of Hsp70-IR cells were seen in the TG of CFA-applied rats (Figure 3A,B, and C). The mean relative number of Hsp70-IR TG neurons was significantly increased on day 3 after CFA application into the M1TP compared with sham or vehicle-applied rats (*n* = 5 in each group) (Figure 2D).

### FG, DiI, and labeled recombinant Hsp70 tracing

We also studied if the TG neurons were retrogradely labeled with both FG and DiI injected into the tongue and M1, respectively (Figure 3E,F,G, and H). About 9.2% (*n* = 5) of TG neurons were labeled with FG and DiI (Figure 3H), indicating that some TG neurons innervate both tongue and M1TP.

We further studied whether fluorescent-labeled recombinant Hsp70 applied to the M1TP was transported to TG neurons in rats with FG injection into the tongue (Figure 3I, J, K, and L). About 15.4% (*n* = 5) of FG-labeled TG neurons were labeled with recombinant Hsp70 (Figure 3L), indicating that fluorescent-labeled recombinant Hsp70 was transported from the M1 tooth pulp to TG neurons and released, and was taken by TG neurons innervating into the tongue.

### Effect of Hsp70 or LPS administration into TG on HWT

**Figure 4:**
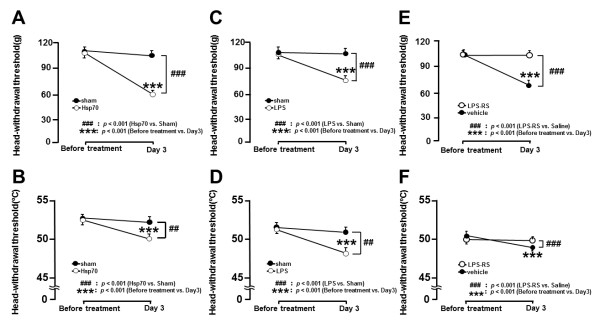
**HWT to mechanical or heat stimulation of the tongue in pulpal Hsp70-, LPS-applied, or sham rats, and the effect of LPS blocker LPS-RS on HWT. A**, **B**: HWT to mechanical **(A)** and heat stimulation **(B)** of the tongue in pulpal Hsp70-applied rats or sham rats. **C**, **D**: HWT to mechanical **(C)** and heat stimulation **(D)** of the tongue in pulpal LPS-applied rats or sham rats. **E**, **F**: Change in HWT to mechanical **(E)** and heat stimulation **(F)** of the tongue following TG injection of LPS-RS or vehicle in pulpal CFA-applied rats. ***, ###: *P* < 0.001.

Three days after recombinant Hsp70 application to the M1TP in naive rats, the HWTs to mechanical and heat stimulation of the tongue were significantly decreased compared with that of saline-applied rats (*P* <0.001, *n* = 5 in each group) (Figure 4A and B).

We also observed significant reduction of the HWT to mechanical and heat stimulation of the tongue at 3 days after LPS application to the M1TP in sham rats compared with saline-applied rats (*P* <0.001, *n* = 5 in each group) (Figure 4C and D).

### Effect of LPS-RS administration into TG on HWT

Following successive LPS-RS administration (0.5 μL/day) for 3 days into TG in M1 CFA-applied rats, the decreased HWTs to mechanical and heat stimulation of the tongue were significantly reversed compared with that of saline-injected rats (*P* <0.001, *n* = 5 in each group) (Figure 4E and F).

### TG neuronal activity

**Figure 5:**
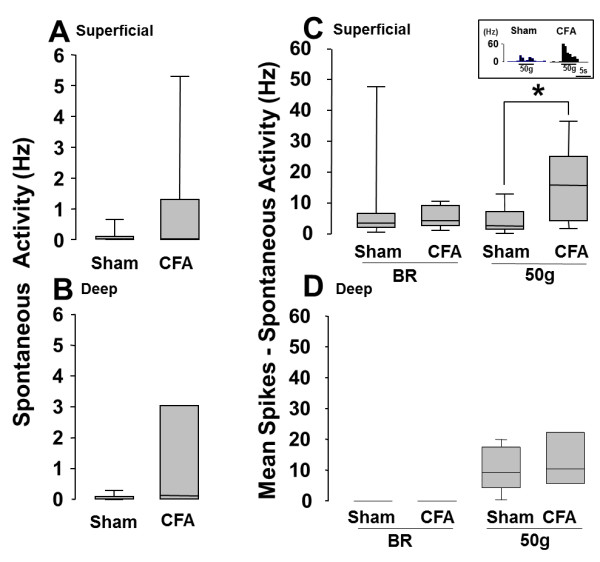
**Spontaneous activities and brush or 50 g noxious mechanical responses of TG nociceptive neurons in M1TP CFA-applied rats or sham rats. A**, **B**: Spontaneous activity of superficial RF neurons **(A)** and deep RF neurons **(B)**, **C**, **D**: Mechanical evoked responses of superficial RF neurons **(C)** and deep RF neurons **(D)**. Sham: M1TP sham-treated rats, inset diagram in **C** indicate typical unit activities of TG neurons, CFA: M1TP CFA-applied rats. *: *P* < 0.05.

To test if TG neurons innervating the tongue increased their activities following CFA application to the M1TP, single neuronal activities innervating the tongue were analyzed in the M1TP-inflamed or sham rats. A total of 26 TG nociceptive neurons innervating the tongue were classified as TG neurons with superficial receptive fields responding to brush (superficial RF neurons; sham, *n* = 9; CFA, *n* = 10) or deep receptive fields responding to brush (deep RF neurons; sham, *n* = 9; CFA, *n* = 8), respectively. No significant changes in spontaneous activities in superficial and deep RF neurons following CFA application to the M1TP (Figure 5A and B). On the other hand, superficial but deep RF neurons showed significant increase in firing frequency following noxious mechanical stimulation of the RFs in M1TP CFA-applied rats compared with sham rats (Figure 5C and D).

### Hsp70 mRNA expression

**Figure 6:**
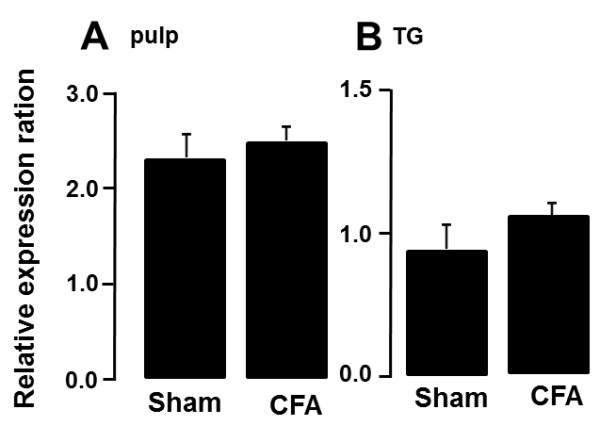
**Relative change in Hsp70 mRNA levels in the M1TP or TG following CFA application to the M1TP or sham treatment of the M1TP. (A)** M1TP; **(B)** TG.

We also studied the change in Hsp70 mRNA level in the M1TP and TG following M1TP inflammation using RT-PCR technique (Figure 6). No significant differences of Hsp70 mRNA levels in the M1TP and TG between CFA-applied rats and sham rats.

## Discussion

This study has provided novel evidence suggesting a role of TLR4 mediating hyperexcitability of TG neurons innervating the non-inflamed tongue resulting in ectopic tongue pain associated with TP inflammation. To evaluate the mechanisms underlying ectopic tongue pain following TP inflammation, measurement of nocifensive reflex and immunohistochemical studies were conducted. We demonstrated that mechanical and heat HWT were significantly reduced, TLR4 and Hsp70 expression in the TG and the excitability of TG nociceptive neurons innervating the tongue were strongly enhanced, Alexa labeled-Hsp70 was transported from the M1TP to TG neurons, and there were some TG neurons innervating both M1TP and tongue. Furthermore, Hsp70 or LPS application to the M1TP caused the decrease in HWT, and LPS-RS to the TG attenuated the enhanced nocifensive reflex.

### TLR4 expression in TG neurons

Previous studies have reported that TLRs expressed in the dorsal ganglion neurons are key molecules involved in modulation of neuronal excitability and respond to endogenous ligands following tissue injury or inflammation [16-18]. Increasing evidence indicates that TLRs and their associated signals contribute to pathological pain conditions, and blockade of TLR signaling has been shown to reduce hyperalgesia [18,19]. It has also been reported that CFA injection into the lower lip causes strong enhancement of the excitability of trigeminal ganglion (TG) neurons innervating non-inflamed upper lip via neuron-neuron interactions [20-22]. We showed the significant enhancement of nocifensive reflex to mechanical and heat stimulation of the tongue following M1TP inflammation, and also observed significant increase in the number of TLR-IR TG neurons innervating the non-inflamed tongue compared with vehicle-applied rats. Together with previous data our results suggest that the TLR4 expressed in TG neurons is a key receptor for ectopic tongue pain associated with M1TP inflammation.

### TLR4 ligands in TG neurons

LPS and Hsp70 are known as the specific ligands for the TLR4, and these molecules bind to the TLR4 expressed in neurons, resulting in the enhancement of neuronal excitability [11,18]. It is also well known that Hsps localize within the cells and bind to heat shock factors, and following an appropriate stress, accumulation of unfolded proteins leads to the dissociation of Hsps from heat shock factors, leaving Hsps free to bind target proteins [23]. On the other hand, LPS is a major cell wall component of Gram-negative bacteria and TLR4 exogenous ligand is recognized by TLR4. We observed strong expression of TLR4 and Hsp70 in TG neurons innervating non-inflamed tongue which is associated with M1TP inflammation. Hsp70 is one of the ligands expressed in inflamed tissues and acts as a ligand for TLR4 [8,23,24]. Following intraganglionic administration of Hsp70 or LPS caused mechanical and heat hyperalgesia in naive rats, and the enhanced mechanical and heat nocifensive reflexes were strongly attenuated by LPS-RS injection to the TG in the rats with M1TP inflammation. Furthermore, we observed significant enhancement of the excitability of TG nociceptive neurons innervating the tongue in M1TP inflamed rats compared with sham rats. These results suggest that Hsp70 expressed in TG neurons binds to TLR4 in TG neurons innervating the tongue and then increases the excitability of those TG neurons following M1TP inflammation.

Based on the previous results and present data, Hsp70 expression after tooth pulp inflammation may have two different functions in accordance with its location; Hsp70 in the tooth pulp exerts neuroprotective, cytoprotective, and housekeeping functions as a chaperone protein, whereas Hsp70 in TG exerts immunomodulatory functions as TLR4 ligands to send peripheral neuroplastic changes or warning signals to the brain.

### Transportation of Hsp70 from M1TP to TG neurons

It has been reported that Hsp70 is expressed in peripheral tissues as well as ganglion cells following tissue inflammation [25]. We hypothesized that Hsp70 was expressed in the M1TP associated with pulpal inflammation and transported from M1TP to TG neurons, or Hsp70 was expressed in TG neurons following pulpal inflammation. To evaluate if Hsp70 is transported to TG neurons following pulpal administration, Alexa-labeled Hsp70 protein was applied to the exposed M1TP in naive rats and analyzed the Alexa labeling in TG neurons. There were many Alexa-labeled neurons in the TG on day 3 after pulpal administration of Alexa-labeled Hsp70, and some of them were also labeled with FG injected into the tongue. We also did not observe differences of Hsp70 mRNA levels in the M1TP and TG between M1TP CFA-applied rats and sham-treated rats. It is likely that Hsp70 is expressed in the inflamed M1TP and transported to the TG neurons with axonal flow or Hsp70 is expressed in the TG neurons innervating in the tongue, and is released from TG neurons innervating the inflamed M1TP. Hsp70 released from TG neurons binds to TLR4, and the neuronal excitability of TG neurons innervating the non-inflamed tongue might be enhanced, resulting in the tongue ectopic pain associated with tooth pulp inflammation.

### TG neurons innervating tooth pulp and tongue

We also observed that 9.2% of TG neurons were retrogradely labeled with FG and DiI injected into the M1TP and tongue, respectively. Our previous studies have reported that about 6% of TG neurons innervate multiple tooth pulps [15]. These suggest that some TG neurons innervate both M1TP and tongue, and this anatomical feature favors the development of ectopic tongue pain associated with M1TP inflammation.

## Conclusions

These results are the first documentation that TLR4 is involved in the ectopic tongue pain associated with M1TP inflammation. Following M1TP inflammation, Hsp70 is expressed in the pulpal tissues, transported to the cell bodies of the trigeminal ganglion neurons, or Hsp70 is expressed in TG cells, and then released from TG neurons innervating the inflamed M1TP. Hsp70 binds to TLR4 in TG neurons innervating the tongue, and the excitability of TG neurons innervating the tongue may be enhanced, resulting in ectopic tongue pain.

## Abbreviations

CFA: Complete freund’s adjuvant; CNS: Central nervous system; DAMPs: Danger-associated molecular patterns; EMG: Electromyogram; FG: Fluorogold; Hsp70: Heat shock protein 70; HWT: Head-withdrawal reflex threshold; i.p.: Intraperitoneal; IR: Immunoreactive; LPS: Lipopolysaccharide; M1: Molar tooth pulp; NGS: Normal goat serum; PAMPs: Pathogen-associated molecular patterns; PNS: Peripheral nervous systems; TG: Trigeminal ganglion; TLR4: Toll-like receptor 4; TLRs: Toll-like receptors; TP: Tooth pulp.

## Competing interests

All authors declare that they have no competing interests.

## Authors’ contributions

KO and KS participated in the design of the experiments, performed the animal studies, analyzed the data, and wrote the manuscript. SM executed the immunohistochemical analyses and revised the manuscript. BO participated in the design of the experiments and revised the manuscript. MA and DO performed the RT-PCR experiments. YT participated in the single neuron recording from TG neurons. MS and KI conceived and designed the study, analyzed the data, and wrote the manuscript. All authors read and approved the final version of the manuscript.
